# Use of patient specific 3D printed neurovascular phantoms to simulate mechanical thrombectomy

**DOI:** 10.1186/s41205-021-00122-8

**Published:** 2021-09-27

**Authors:** Kelsey N. Sommer, Mohammad Mahdi Shiraz Bhurwani, Vincent Tutino, Adnan Siddiqui, Jason Davies, Kenneth Snyder, Elad Levy, Maxim Mokin, Ciprian N. Ionita

**Affiliations:** 1 Department of Biomedical Engineering, University at Buffalo, Buffalo, NY 14228 USA; 2 Canon Stroke and Vascular Research Center, University at Buffalo, Buffalo, NY 14208 USA; 3 Department of Neurosurgery, University at Buffalo, Buffalo, NY 14208 USA; 4 Department of Pathology and Anatomical Sciences, University at Buffalo, Buffalo, 14208 USA; 5 Department of Biomedical Informatics, University at Buffalo, Buffalo, 14208 USA; 6 Department of Neurosurgery and Brain Repair, University of South Florida, Tampa, FL 33620 USA

**Keywords:** Mechanical thrombectomy, 3D printing, Large vessel occlusion, Thrombolysis in cerebral infarction

## Abstract

**Background:**

The ability of the patient specific 3D printed neurovascular phantoms to accurately replicate the anatomy and hemodynamics of the chronic neurovascular diseases has been demonstrated by many studies. Acute occurrences, however, may still require further development and investigation and therefore we studied acute ischemic stroke (AIS). The efficacy of endovascular procedures such as mechanical thrombectomy (MT) for the treatment of large vessel occlusion (LVO), can be improved by testing the performance of thrombectomy devices and techniques using patient specific 3D printed neurovascular models.

**Methods:**

3D printed phantoms were connected to a flow loop with physiologically relevant flow conditions, including input flow rate and fluid temperature. A simulated blood clot was introduced into the model and placed in the proximal Middle Cerebral Artery (MCA) region. Clot location, composition, length, and arterial angulation were varied and MTs were simulated using stent retrievers. Device placement relative to the clot and the outcome of the thrombectomy were recorded for each situation. Digital subtraction angiograms (DSA) were captured before and after LVO simulation. Recanalization outcome was evaluated using DSA as either ‘no recanalization’ or ‘recanalization’. Forty-two 3DP neurovascular phantom benchtop experiments were performed.

**Results:**

Clot angulation within the MCA region had the most significant impact on the MT outcome, with a *p*-value of 0.016. Other factors such as clot location, clot composition, and clot length correlated weakly with the MT outcome.

**Conclusions:**

This project allowed us to gain knowledge of how such characteristics influence thrombectomy success and can be used in making clinical decisions when planning the procedure and selecting specific thrombectomy tools and approaches.

## Background

Three-Dimensional printing (3DP) offers the ability to build geometrically-accurate patient-specific vascular phantoms/models that can aid clinical decision making, including treatment planning [1]. In addition, these phantoms may be used for benchtop experimentation, device testing, and physiological simulations for hemodynamics investigation and complex fluid-device structure interactions assessments [2–5]. Current multi-material printers replicate complex human vascular anatomy into a photopolymer replica within a few tens of microns accuracy, using materials that mimic vascular mechanical properties, thus allowing realistic simulations of endovascular interventions [6, 7]. Use of these phantoms, to practice various approaches and procedures shows promise as a method to optimize interventional outcomes and reduce the rate of peri-procedural complications.

3D printing has shown much promise in simulating vascular procedures with chronic conditions associated to them including ischemia, myocardial infarction, abdominal aortic aneurysms, and arteriovenous malformation [7–11]. Use of these phantoms to practice various approaches has been proven as a method to improve interventional outcomes, reduce the risk of periprocedural complications, and optimize treatment planning. The ability to mimic the arterial wall mechanical properties such as compliance and stiffness [3, 4, 12–14] in combination with programmable pumps to replicate cardiac waveforms and controlled outflow systems that mimic the capillary resistance [15, 16], allows for creation of comprehensive systems that provide means to simulate the local and global hemodynamics.

On the other hand, simulation of acute conditions including acute ischemic stroke (AIS) from large vessel occlusion (LVO) using 3D printed patient specific phantoms has not been fully investigated. These kinds of studies would be beneficial in providing insight to the clinicians regarding the various techniques and devices that are used for acute treatment of stroke which affects nearly 700,000 people in the United States annually. Often, the cause of these strokes is a lack of blood flow due to an arterial LVO in need of endovascular revascularization using stent retriever mechanical thrombectomy (MT) [17, 18]. During these procedures a retrievable device is deployed across the clot which becomes entrapped within the wiring of the device and is subsequently removed by retrieving the device. Stent retriever thrombectomy is currently recommended in patients with AIS from LVO. The success of thrombectomy is graded using Thrombolysis in Cerebral Infarction (TICI) scale which ranges from 0 (full occlusion) to 3 (no occlusion). Recanalization is the main factor that determines whether the treatment method of thrombectomy of AIS patients with LVO produced a good treatment outcome [19, 20]. If successful recanalization is achieved, the patient is 4–5 times more likely to recover with minimal disability after stroke.

For this study we propose to develop patient specific 3D printed models which allow AIS simulation and subsequent mechanical thrombectomy while using flow conditions relevant to cerebral hemodynamics. Using this setup, we also propose to study how variants, such as clot consistency, location and local geometry affect the efficacy of endovascular procedures for the treatment of LVOs. This type of study could add significant knowledge in regards to MT technique comparisons [14, 21, 22].

## Methods

### Patient specific model design

This study was approved by the IRB at University at Buffalo. We used retrospectively-collected data of patients who underwent CT-angiography and had a lesion free main cerebral vasculature. Patients underwent 320- detector row CT angiography (Aquilion ONE, Canon Medical Systems, Tustin, CA). The basilar arteries, internal carotid, vertebral, as well as the Circle of Willis, middle cerebral arteries (MCA), anterior cerebral arteries (ACA), and posterior cerebral arteries (PCA) were segmented using a Vitrea workstation (Vital Images, Minnetonka, MN) with a voxel size of 0.625 × 0.625 × 0.5 mm and a slice thickness of 0.5 mm. Stereolithographic (STL) files were saved of the patient geometry and imported in Autodesk Meshmixer, an advanced mesh manipulation software (San Rafael, California). The neurovascular section of the phantom contains arteries distal to the external carotid artery bifurcation, extending to the second bifurcation in the middle cerebral, and anterior communicating arteries. For this vascular domain the vessel diameters ranged from approximately 2 mm to 5 mm, properly correlating to the human anatomy [23].

**Fig. 1:**
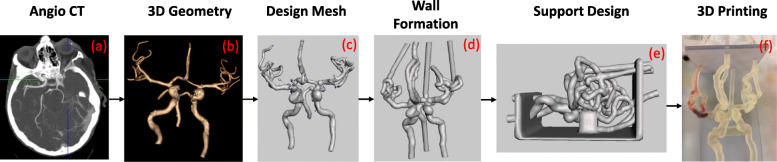
Flow chart of images describing the manufacturing process for a patient specific phantom of the Circle of Willis. **a** An Angio CT image is acquired of the neurovasculature. **b** The neurovasculature is segmented out from the rest of the brain tissue and a 3D geometry is created. **c** A 3D mesh of triangular vertices is created within Autodesk Meshmixer. **d** The mesh is made a solid geometry and hollowed out for the creation of vessel lumens and a (**e**) support structure holds in place the vessels. Arrow pointing to hardened support material to reduce vessel sagging. **f** The model is 3D printed in Stratasys Tango+ material to simulate the vascular compliance and is ready to be connected to a flow loop for simulation studies

The phantom manufacturing process including the 3D mathematical operators used to design the phantom, have been explained in full detail in previous work [2, 5, 24, 25] and design steps will be only briefly described (Fig. 1). Within Meshmixer, lumen segmentation artifacts were removed and a minimal smoothing process reduced the number of artifacts while maintaining the overall geometry of the vasculature. A base designed in SolidWorks (SolidWorks Corp., Waltham, MA) was appended to the vasculature as a support structure to provide stability to the phantom during the benchtop flow experimentation. In addition, the ICA segment between the carotid siphon and ophthalmic artery segment was either supported or reinforced by using a hardened material (Fig. 1(e) arrow) to reduce any sagging along the curvature of the vessel for proper device navigation. The phantom was 3D printed in a soft material, Stratasys Tango+ (Stratasys, Eden Prairie, MN) to replicate the neurovascular wall elasticity [12].

Optimal post-print processing of the inner artery lumen to achieve realistic device artery surface interactions, a friction analysis was conducted in a previous study [12, 26] Using their findings, we implemented a sodium hydroxide solution to etch the rough inner surfaces followed by coating with HidroMed (AdvanSource Biomaterials Wilmington, MA). Wall thickness and material composition were both tested to replicate the compliance of the neurovasculature. A compliance chamber was developed to measure changes in vessel diameter under pressures ranging from 0 to 210 mmHg while the vessels were submerged in body temperature water to simulate physiological conditions. The results obtained concluded that Tango+ with a 1 mm thickness and 4.5 mm diameter was the only material to exhibit a compliance close to the healthy range (0.08–0.12 mm2/mmHg) of 0.075 mm2/mmHg.

The accuracy of the 3D printed models were also tested within another study performed within our group prior to this experimentation [4]. The centerlines from both of the CCTA images of the patient and the phantom were generated within Mimics Research (Materialize, Plymouth, MI) and the minimum diameter, maximum diameter, best fit diameter, cross-sectional area, and tortuosity were calculated. It was concluded that the phantom diameter measurements were within 1 mm of the patient images on average and the tortuosity had a very small average difference. This verifies that our 3D printed phantoms created in an elastic material are maintaining the three-dimensional geometry.

### Benchtop flow experimentation

**Fig. 2:**
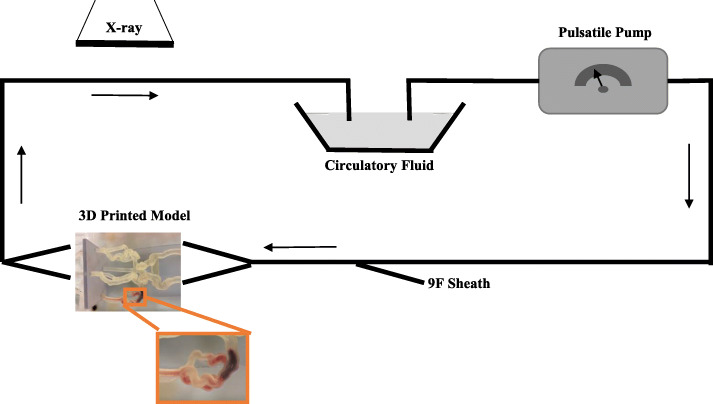
Schematic representation of the clot model. The model contains separate inflow and outflow channels and is connected to a pulsatile pump via a closed circuit. Arrows indicate direction of flow. A 9 F sheath allows the introduction of guide catheters and thrombectomy devices. Biplane angiography is used during thrombectomy experiments. A zoomed in image of the 3D printed model displays a clot located within the M2

Phantoms were connected to a flow loop with a simulated physiologically relevant input flow rate of the carotid artery and fluid temperature of 37 degrees Celsius (Fig. 2) [27]. We maintained the temperature of the fluid within the flow loop to be consistently at body temperature using an Anova sous vide (Anova Applied Electronics, Inc. San Francisco, CA) as the Nitinol used in the clot retriever devices is strongly dependent on temperature. Standard digitally subtracted angiograms (Canon Medical Systems Corp., Tustin, CA) were taken with a Canon Infix C-arm prior to insertion of the clot and medical devices. DSA images were obtained at 10 frames/sec at system-selected parameters of kV and mA. Fresh clots were prepared following the methods presented by Duffy, et.al [28].. Clot type D (40% red blood cells, calcium chloride) and type G (pure fibrin, calcium chloride) were created.

Clots were then measured into pieces of varying lengths between 5 mm and 25 mm for the experimentation.

**Fig. 3:**
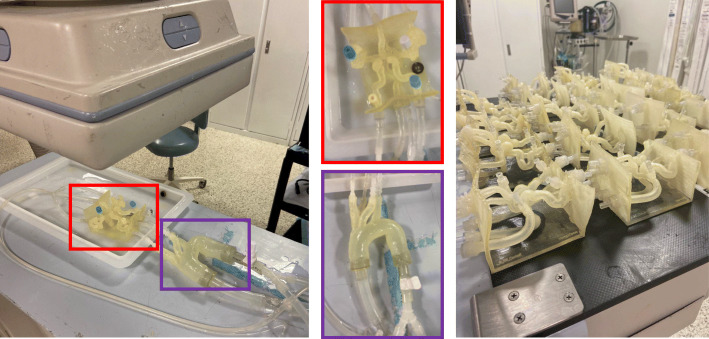
The 3D printed patient specific Circle of Willis is connected to a flow loop. (Red) The patient specific neurovasculature and (Purple) a standard aortic arch are highlighted. 20 different patient specific models have been printed. A clot is introduced into the proximal MCA and tests the effectiveness of stent retriever thrombectomy with TICI scoring system in patients with absent or robust collaterals of the circle of Willis using a conventional vs. BGC

**Fig. 4:**
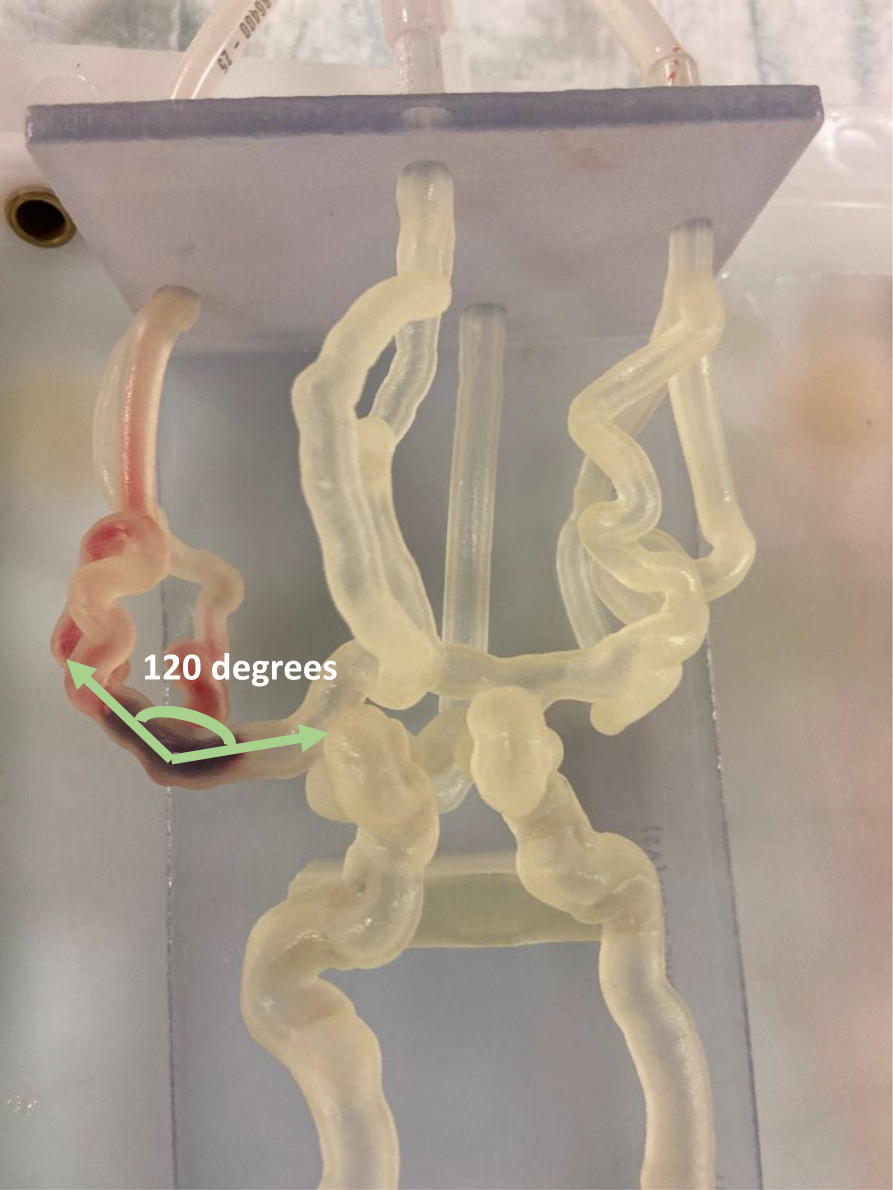
The angulation of the clot within the vasculature was measured as an experimental variable to determine if it has effect on the experimental outcome

A clot was introduced into the model and placed anywhere in the M1 or M2. The 3D printed patient specific models connected to a flow loop is shown in Fig. 3. A guide catheter was inserted into the internal carotid artery (ICA) of the 3D printed phantom on the side of occlusion. The guide catheter provides support for the microcatheter which was next inserted to navigate to the location of the occlusion. The stent retriever was deployed across the simulated blood clot at the occlusion site. We used both Trevo XP (Stryker Corporation, Kalamazoo, MI) and Solitaire X (Medtronic, Dublin, Ireland) stent retrievers. Stent retriever diameters ranged from 4 to 6 mm and did not influence the experimental outcome so we did not include those results in this study. A stent retriever thrombectomy was then simulated with the following parameters studied: angulation (angle of the vasculature in which the clot is placed) (Fig. 4), clot length (before and after insertion), clot morphology (D/G clots), clot location within the device and within the vasculature, and treatment approach (standard thrombectomy with/without aspiration). DSA was performed prior to thrombectomy to confirm adequate occlusion and after thrombectomy to document angiographic outcome. DSA was graded according to the TICI scale. TICI 2b/3 was considered “successful” recanalization, TICI 0-2a was consider “unsuccessful” recanalization.

### Statistical analysis

A *p*-value < 0.05 was considered indicative of a statistically significant difference. A multi-variable regression model was performed on the entirety of the data with the experimental outcome as the y-variable and the clot angulation within the vasculature (x_1_), initial clot length (x_2_), clot composition (x_3_), clot location within device (x_4_), and clot location within vasculature (x_5_) as the x-variables. Estimated coefficients (β_0_, β_1_, β_2_, β_3_, β_4_, β_5_), *p*-values and odds ratios were determined. Odds ratios were determined by binarizing the results as follows: clot angulation (> = 120°, < 120°); clot length (> 12 mm, <=12 mm); clot composition (hard, soft); clot location in device (mid, proximal/distal); clot location in vasculature (M1/M2).

## Results

**Fig. 5:**
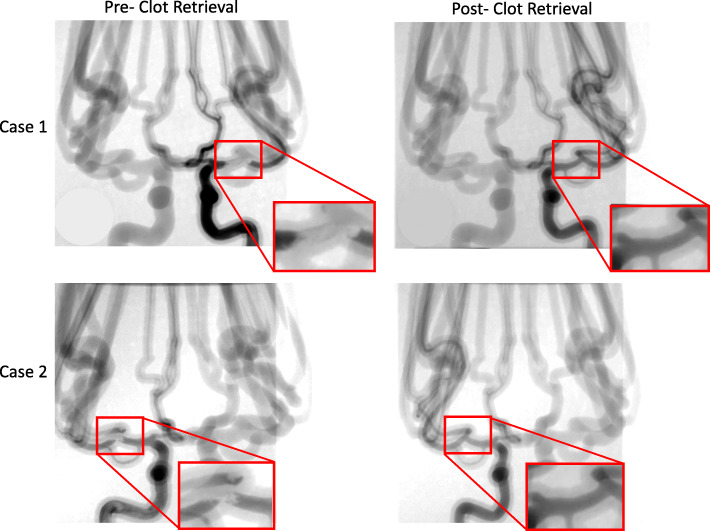
Angiograms were taken both pre- and post- stent retriever thrombectomy was completed. Pre- clot retrieval, there is very little or no contrast flowing at the location of the clot. Post- clot retrieval there is contrast flowing through the part of the vessel where the clot was removed. Case 1 and case 2 in this figure display this significant change in fluidic flow at the location of the clot. The red boxes are enlarged views of the specific locations where the contrast flow changes

**Fig. 6:**
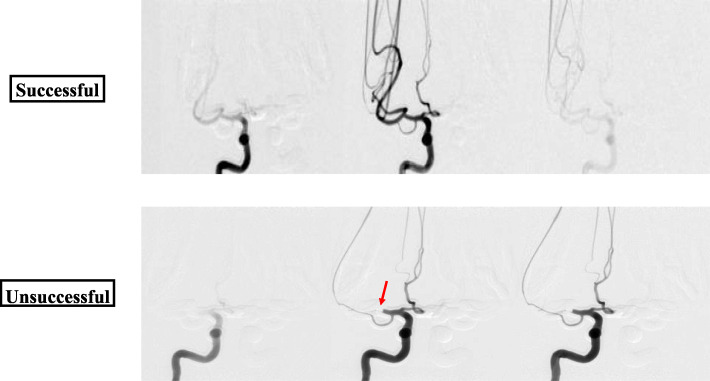
Angiograms were taken post-stent retriever thrombectomy for each of the cases. A successful case and an unsuccessful case are displayed above. The successful case shows contrast flowing through the neurovascular phantom resulting in no occlusion. While the unsuccessful case shows the contrast flow being halted where the blood clot is still blocking the vessel resulting in full occlusion (red arrow)

Using these phantoms, angiograms were captured before recanalization, showing the extent of the thrombus, and after the thrombectomy to determine the recanalization outcome. Figure 5 displays the change in blood flow before and after the stent retriever has been deployed. Figure 6 depicts a montage of the contrast flowing through a 3D printed model after both an unsuccessful and a successful mechanical thrombectomy simulated procedure.

**Fig. 7:**
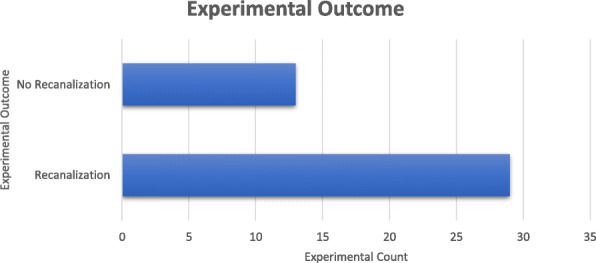
An experimental outcome was determined for each of the 42 benchtop experiments performed as ‘No Recanalization’ or ‘Recanalization’

The experimental outcome was recorded for each experiment as either recanalization or no recanalization (Fig. 7). Based on our results, 29 of the 42 benchtop experiments were successful.

The results for the 42 experiments have been analyzed based on the parameters we changed within the study to determine their single variable significance to experimental outcome (Table 1).

**Table 1:** Single Variable Statistical Tests of Significance were determined for each of the following experimental variables: clot angulation, clot length, clot composition, clot location in device, and clot location in vasculature. Either a student t-test or a chi-squared test were performed to output the *p*-value for each experimental variable. Means for both successful and unsuccessful cases are presented with ±95% confidence

Experimental Variable	Statistical Test	Mean (Successful)	Mean (Unsuccessful)	***P***-Value
**Clot Angulation**	Student T-test	118.10 ± 19.44°	153.46 ± 17.37°	0.016
**Clot Length**	Student T-test	16.76 ± 2.64 mm	15.38 ± 3.68 mm	0.557
**Clot Composition**	Chi-Squared Test	N/A	N/A	0.115
**Clot Location in Device**	Chi-Squared Test	N/A	N/A	0.196
**Clot Location in Vasculature**	Chi-Squared Test	N/A	N/A	0.579

Based on these experimental parameters, the ‘Clot Angulation’ proved to be the only experimental variable of ‘Significance’ with a *p*-value of 0.016. Table 1 also displays the means with 95% confidence intervals of each experimental variable for both the successful and unsuccessful experimental outcomes.

Based on the multi-variable regression model used, estimated regression coefficients were output for each experimental independent variable to determine whether they have an impact on the experimental outcome (Table 2). All estimated regression coefficients were very close to zero which implies that all the experimental variables have little impact on the experimental outcome.

**Table 2:** Multi-variable statistical analysis was performed on the experimental data in which clot angulation (x_1_), clot length (x_2_), clot composition (x_3_), clot location in device (x_4_), and clot location in vasculature (x_5_) were allocated to be the model predictors. Regression coefficients, R^2^, adjusted R^2^, and an overall p-value were output from the model

Model: y = β_**0**_ + x_**1**_β_**1**_ + x_**2**_β_**2**_ + x_**3**_β_**3**_ + x_**4**_β_**4**_ + x_**5**_β_**5**_				
Predictor	Regression Coefficient	Estimate	P-Value	Odds Ratio (95% Confidence Intervals)
**Constant**	β_0_	1.005	0.0002	
**Clot Angulation (x**_**1**_**)**	β_1_	−0.140	0.005	0.170 (0.032–0.905)
**Clot Length (x**_**2**_**)**	β_2_	0.176	0.820	1.828 (0.457–7.315)
**Clot Composition (x**_**3**_**)**	β_3_	−0.183	0.369	1.300 (0.275–6.137)
**Clot Location in Device (x**_**4**_**)**	β_4_	−0.008	0.179	0.885 (0.232–3.380)
**Clot Location in Vasculature (x**_**5**_**)**	β_5_	−0.004	0.664	1.714 (0.452–6.506)

Odds ratios were determined by binarizing the results as follows: clot angulation (> = 120°, < 120°); clot length (> 12 mm, <=12 mm); clot composition (hard, soft); clot location in device (mid, proximal/distal); clot location in vasculature (M1/M2)

## Discussion

3D printing offers a unique opportunity to build geometrically accurate patient specific vascular phantoms that can be used for benchtop testing, flow simulations, treatment planning, device testing, and physiological simulations. Patient specific vascular models engineered through additive manufacturing can be used to visualize complex anatomical structures and simulate device deployment. Previous studies have used phantoms with stiff photopolymers that lack the compliance of the vasculature which is crucial for properly simulating the physiology within the vascular anatomy [15, 29–31]. To capture the compliant nature of the vasculature, flexible photoresins such as the Stratasys Tango family (Stratasys, Eden Prairie, MN) and the Visijet 3D Systems family (3D Systems, Rock Hill, SC) is needed and has been tested under previous investigation [26]. With the preservation of the hemodynamics within the vasculature by means of compliance, stiffness and pressure simulations, 3D printed vascular models can accurately depict the fluid mechanics within the human anatomy [32–34].

We performed a comprehensive study using 3D printed patient specific neurovascular phantoms which may allow for a relatively simple means to test devices, fine tune procedures, and train surgeons. This project employs a novel approach that combines 3D model manufacturing technology with the ability to generate patient-specific anatomical variants for accurate simulation of real-world clinical scenarios of AIS from LVO treated with mechanical thrombectomy.

Through the evaluation of controlled changing parameters within our experimental setup, we were able to demonstrate the use of 3DP vascular phantoms to simulate acute complications such as AIS. In addition, we design a set of tests to determine single variable and multivariable significance on the experimental outcome using variants that may affect the MT such as clot composition, location, geometry and length. The local geometry, namely the clot angulation, proved to be the single significant experimental variable, *p*-value of 0.016, that affects the experimental thrombectomy outcome. Acute angles may prevent the device from fully engaging the clot, and thus reduce the thrombectomy effectiveness. Clot length within our testing range, 5 mm to 25 mm, was not a significant factor which might also be due to the fact that the devices used were between 30 and 40 mm long and enclosed fully the device. Also, the longer clots tend to stop more proximal in the circle of Willis which made them easier to access and remove. This last aspect also ties into the location analysis, we did not see a significant correlation between the location within the vasculature and MT efficacy.

There are a few phantom design related limitations to our study. 3D printing based phantoms are subject to CT imaging errors including scan errors due to patient motion, blooming due to calcification, and a reduction in image accuracy with the 3DP resolution of 200 μm surpassing the spatial resolution of the CCTA of 630 μm. Since most of the neurovasculature are between 2 to 4-mm diameter, small segmentation errors can result in significant changes in the hemodynamics. Involving the segmentation process, there may have been some errors in defining the vessel wall boundaries with high accuracy; however, we cross-validated this process between two users to avoid significant errors. Also, in the process of segmentation artifact elimination, we sculpted the mesh within Autodesk Meshmixer manipulating single triangular vertices, which might have created slight geometric alterations. Additionally, with the models being printed in an elastic material, this allows for deformations to occur within the printing process which may affect the accuracy of the benchtop testing over time.

Other limitations are related to the proposed benchtop system. Since the experimental implementation was challenging due to accumulation of iodinated contrast and clot fragments we did not use a standard water glycerol mixture to simulate blood viscosity. This is a limitation of our study since use of water only as a working fluid could potentially change the fluid dynamics of the system especially at the occlusion location, due to the complexity of the simulation and the abundance of unknown variables being tested. We heated the water to body temperature as the nitinol within the thrombectomy devices reacts best at this temperature. However; the clot to vessel wall interaction may introduce error into the system as the composition of both surfaces vary from that of the human blood clots and vessels.

As an in vitro-study our approach has some limitations but also benefits which may not be replicated with other in-vitro models or animal models. The limitations include, only partial reproducibility of the biomechanical properties of the arterial network and device-clot-blood interaction and missing the effect of distal microvasculature which could affect the hemodynamics. On the other hand, the 3D printing models allow studies to be performed in an identical replica of the human vasculature, using patients who underwent mechanical thrombectomies with known outcomes. This fact may become far more relevant for device testing then animal models or idealized models since it allows not only to research generic device behavior in human like geometries but also optimize this kind of a treatment.. There is also great value of this benchtop setup as a training device for interventionalists. Before training on a human patient, these patient specific 3DP printed models incorporated into our benchtop set up allow for techniques such as stent retriever deployment, catheter insertion, and image-guided device navigation. With the incorporation of vessel elasticity and compliance parameters, these models provide a novel approach in simulated patient specific vascular studies.

## Conclusions

The main advantage of using this in vitro model of thrombectomy is that it provides a highly controlled environment where only a single variable (such as angulation of MCA or clot length) or treatment approach can be changed at a time. This project allowed us to gain knowledge of how such characteristics influence thrombectomy success can be used in making clinical decisions when planning the procedure and selecting specific thrombectomy tools and approaches.

## Data Availability

The datasets used and/or analyzed during the current study are available from the corresponding author on reasonable request.

## Abbreviations

3DP: Three-Dimensional Printing
MT: Mechanical Thrombectomy
AIS: Acute Ischemic Stroke
LVO: Large Vessel Occlusion
MCA: Middle Cerebral Artery
DSA: Digital Subtraction Angiography
